# Development and validation of a nomogram for predicting pulmonary embolism in patients with non-small cell lung cancer

**DOI:** 10.12669/pjms.41.12.13232

**Published:** 2025-12

**Authors:** Xiaoting Wu, Shanshan Tang

**Affiliations:** 1 Xiaoting Wu, Department of Respiratory and Critical Care, First People’s Hospital of Linping District, Hangzhou, Zhejiang Province 311199, P.R. China; 2 Shanshan Tang, Department of Respiratory and Critical Care, First People’s Hospital of Linping District, Hangzhou, Zhejiang Province 311199, P.R. China

**Keywords:** Non-small cell lung cancer, Pulmonary embolism, Risk prediction, Nomogram model

## Abstract

**Objective:**

In patients with non-small cell lung cancer (NSCLC) complicated by pulmonary embolism (PE), the clinical manifestations become more complex and the diagnosis is more difficult. We aimed to develop and validate an individualized nomogram for differentiating the PE of NSCLC.

**Methodology:**

Patients with NSCLC at the First People’s Hospital of Linping District, Hangzhou were enrolled from September 2021 to March 2024. Least Absolute Shrinkage and Selection Operator (LASSO) and multivariate logistic regression analyses were performed to recognize risk factors. An individualized nomogram was subsequently developed. The model’s performance was validated using the receiver operating characteristic (ROC) curve, calibration plot, and decision curve analysis (DCA).

**Results:**

We enrolled 390 NSCLC patients, of whom 89 (22.8%) had PE. Using multivariate logistic regression, we finally identified seven independent risk factors for PE: pathological type, tumor-node-metastasis (TNM) staging, indwelling central venous catheter (CVC), chemotherapy, hemoglobin, white blood cell count (WBC), and neutrophil-to-lymphocyte ratio (NLR). The model showed good predictive ability, with an area under the ROC curve of 0.909 (95% CI: 0.875–0.942). The calibration curves of the model showed good agreement between actual and predicted probabilities. The ROC and DCA curves demonstrated that the nomogram exhibited a good predictive performance.

**Conclusions:**

The nomogram model for predicting the risk of PE in NSCLC has good predictive performance and is potentially useful for screening of high-risk patients in clinical practice.

## INTRODUCTION

Lung cancer, particularly non-small cell lung cancer (NSCLC), is one of the malignancies with the fastest-growing incidence and mortality rates, posing a significant threat to human health and life.^1^,^2^ NSCLC is a malignant tumor that originates from the bronchial mucosa or glands of the lungs.^1^ Pulmonary embolism (PE) refers to the collective term for diseases or clinical syndromes caused by various types of thrombosis obstructing the pulmonary artery or its branches, with thrombosis being the most common type of embolism.^3^ Research findings indicate that there is a potential association between cancer and the occurrence of venous thromboembolism.^4^ As one of the most severe manifestations of venous thromboembolism, PE is a common complication of malignant tumors.^5^ However, the symptoms and signs of NSCLC combined with PE do not show specific clinical features compared with NSCLC alone. The characteristic features of PE become less recognizable due to its earlier onset, leading to undiagnosed or delayed diagnoses.^4^ The complex mechanisms underlying the NSCLC combined with PE resulted from the combined influence of such as age, sex, genetic factors, NSCLC pathological type and tumor node metastasis staging, and treatments including surgery, chemotherapy, and radiotherapy.^6^-^8^ Currently, most studies are based on the analysis of risk factors for patients with NSCLC combined with PE, therefore, there is an urgent need to develop a predictive model for individualized risk assessment.

By integrating a variety of prognostic and determinant variables to generate an individual probability of a clinical event, nomogram meet our need for models that combine biological and clinical risks.^9^ Notably, few studies have focused on the development and implementation of clinical prediction nomogram for patients with NSCLC combined with PE. In this study, a nomogram was established for prediction retrieved from the First People’s Hospital of Linping District, Hangzhou in hopes of elucidating further clinical practice.

## METHODOLOGY

This retrospective study reviewed the data of 390 patients admitted to the respiratory and critical care inpatient unit of the First People’s Hospital of Linping District, Hangzhou from September 2021 to March 2024. Eligible patients were eighteen years old or above, had a confirmed histopathological diagnosis of NSCLC, and underwent a computed tomography pulmonary angiography combined with clinical symptoms and indicators which could confirm the presence or absence of PE at the time of NSCLC diagnosis.^10^

### Ethical approval:

*It* was obtained from the Ethics Committee of the First People’s Hospital of Linping District (Ref# 2023-040; Date: October 9, 2023).

### Exclusion criteria:

- Patients had other known malignant tumors apart from NSCLC;
- Had a history of thrombotic disease;
- Combined with immune system disease, mental disease, or coagulation dysfunction;
- Had an incomplete clinical data.

### Data collection methods:

Following approval by the institutional review board to assess patient records, we collected the following variables based on electronic case records and laboratory testing system. The clinical parameters included age, gender, body mass index (BMI, overweight considered as BMI>25 kg/m^2^), smoking history (at least one pack of cigarettes per day and lasting for six months), alcohol consumption history (consuming alcohol at least once per week and more than one month), and comorbidities such as chronic obstructive pulmonary disease (COPD) and hypertension. Additional clinical variables and the laboratory tests were collected prior to diagnosed as NSCLC or PE with NSCLC, measuring the following parameters: surgery, pathological type, TNM staging, indwelling central venous catheter (CVC), chemotherapy, hemoglobin, D-dimer (D-D), neutrophil-to-lymphocyte ratio (NLR), platelet-to-lymphocyte ratio (PLR), monocyte-to-lymphocyte ratio (MLR), and white blood cell count (WBC).

### Construction and validation of the nomogram prediction model:

The least absolute shrinkage and selection operator (LASSO) regression analysis was performed to screen variables of NSCLC in patients with PE. Then, multivariate logistic regression analysis was constructed based on the independent variables identified by the LASSO. Subsequently, the model included independent variables of statistical difference was visualized as a nomogram and comprehensively evaluated by C-index, the corrected C-index, receiver operating characteristic (ROC) curves, calibration curves, and clinical decision curves.

### Statistical analysis:

Statistical analysis was performed using R program (Version 4.3.0). Categorical variables were presented as counts with percentages, and the proportions were evaluated using the chi-square test. According to the Shapiro-Wilk normality test for assessing the normality of continuous variables, non-normally distributed variables were presented as median (25th and 75th quartiles), and the Wilcoxon rank-sum test was utilized to investigate statistical differences. To identify the independent variables associated with PE with NSCLC, LASSO regression analysis was performed. A multivariable logistic regression model was subsequently developed. The performance of this model was evaluated using the C-index, an optimism-corrected C-index derived through bootstrapping, ROC curves, calibration plots, and decision curve analysis (DCA). All statistical tests were two-tailed, with a *P*-value threshold of < 0.05 for determining statistical significance.

## RESULTS

We included 390 eligible patients with NSCLC, of whom 258 were male and 132 were female. The age of the patients ranged from 46 to 72 years, with a median age of 60 (54-65) years. 89 of them had pulmonary embolism. As illustrated in Table-I, there were no statistically significant differences (*P* > 0.05) between the PE and non-PE groups regarding gender, age, BMI, comorbidities (including COPD and hypertension), smoking and alcohol consumption history, surgery, and PLR. In terms of pathological type, TNM stage, indwelling CVC, chemotherapy, hemoglobin, WBC, and NLR, statistically significant differences (*P* < 0.05) were observed.

**Table-I:** Table 1 Basic clinical characteristics of the PE group and the non-PE group

Characteristics	Overall	Non-PE group	PE group	*P*-value
N	390	301	89	
Age, years				
<60	123 (31.5)	95 (31.6)	28 (31.5)	0.986
≥ 60	267 (68.5)	206 (68.4)	61 (68.5)	
Gender (male)	258 (66.2)	195 (64.8)	63 (70.8)	0.293
BMI, kg/m*2*				
<25	168 (43.1)	129 (42.9)	39 (43.8)	0.872
≥25	222 (56.9)	172 (57.1)	50 (56.2)	
Hypertension, yes	149 (38.2)	120 (39.9)	29 (32.6)	0.214
COPD, yes	119 (30.5)	89 (29.6)	30 (33.7)	0.456
Smoking history, yes	211 (54.1)	161 (53.5)	50 (56.2)	0.654
Drinking history, yes	206 (52.8)	163 (54.2)	43 (48.3)	0.332
Surgery, yes	120 (30.8)	91 (30.2)	29 (32.6)	0.673
Chemotherapy, yes	120 (30.8)	71 (23.6)	49 (55.1)	<0.001
Pathological type				
Adenocarcinoma	227 (58.2)	158 (52.5)	69 (77.5)	<0.001
Non-adenocarcinoma	163 (41.8)	143 (47.5)	20 (22.5)	
TNM staging				
I-II	213 (54.6)	185 (61.5)	28 (31.5)	<0.001
III-IV	177 (45.4)	116 (38.5)	61 (68.5)	
Indwelling CVC, yes	37 (9.5)	15 (5.0)	22 (24.7)	<0.001
Hemoglobin, g/L				
<140	174 (44.6)	115 (38.2)	59 (66.3)	<0.001
≥140	216 (55.4)	186 (61.8)	30 (33.7)	
D-D, µg/mL	1.12 [0.73, 1.26]	1.11 [0.71, 1.26]	1.14 [0.94, 1.57]	<0.001
WBC x10/L	8.30 [7.04, 9.10]	7.86 [6.82, 8.64]	9.10 [8.68, 9.99]	<0.001
PLR	154.00 [136.25, 165.00]	154.00 [136.00, 165.00]	158.00 [147.00, 174.00]	0.090
NLR	6.28 [3.51, 6.64]	6.20 [3.43, 6.60]	6.32 [6.02, 6.97]	<0.001
MLR	0.64 [0.39, 0.69]	0.65 [0.38, 0.69]	0.53 [0.47, 0.81]	<0.001

***Note:*** Values are presented as number (%), or median (interquartile range) ***Abbreviations:*** NSCLC non-small cell lung cancer, PE pulmonary embolism, COPD chronic obstructive pulmonary disease, TNM tumor node metastasis, CVC central venous catheter, D-D D-dimer, NLR neutrophil-to-lymphocyte ratio, PLR platelet-to-lymphocyte ratio, MLR monocyte-to-lymphocyte ratio, WBC white blood cell count.

**Fig.1:**
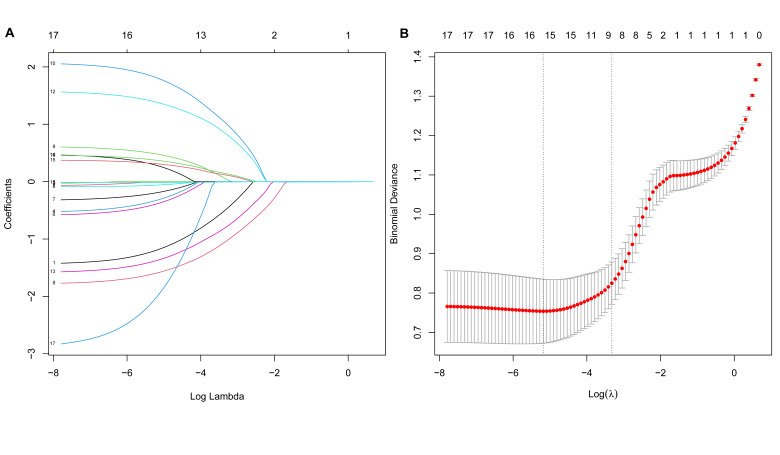
LASSO regression analysis with 10-fold cross-validation for candidate variable identification. (A) A plot of the LASSO coefficient. (B) 10-fold cross-validation curve. LASSO, least absolute shrinkage and selection operator.

**Fig.2:**
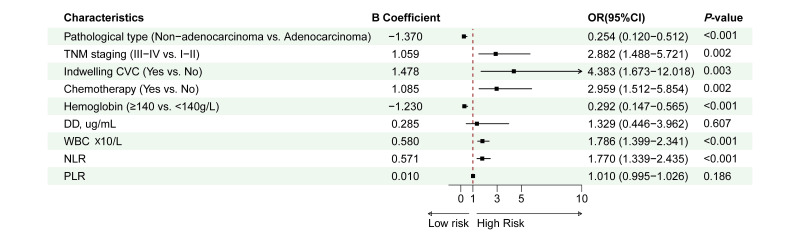
Forest plot of multivariable logistic regression model.

It was depicted in the LASSO path diagram that with the decrease in coefficients, there was a corresponding reduction in the number of predictors (Fig.1A). Subsequently, nine predictor variables were selected based on lambda.1se in Fig.1B: pathological type, TNM stage, indwelling CVC, chemotherapy, hemoglobin, D-D, WBC, NLR, and PLR. Then, a multivariate logistic regression analysis was performed on the above variables, which was presented in Fig.2. We found that the occurrence of PE was associated with III−IV TNM staging (OR=2.88; 95%CI 1.49-5.72), indwelling CVC (OR=4.38; 95%CI 1.67-12.02), chemotherapy (OR=2.96; 95%CI 1.51-5.85), high WBC (OR=1.79; 95%CI 1.40-2.34) and NLR (OR=1.77; 95%CI 1.34-2.44) levels. However, patients with non−adenocarcinoma (OR=0.25; 95%CI 0.12-0.51) and high hemoglobin (≥140g/L) (OR=0.29; 95%CI 0.15-0.57) were less likely to develop PE.

**Fig.3:**
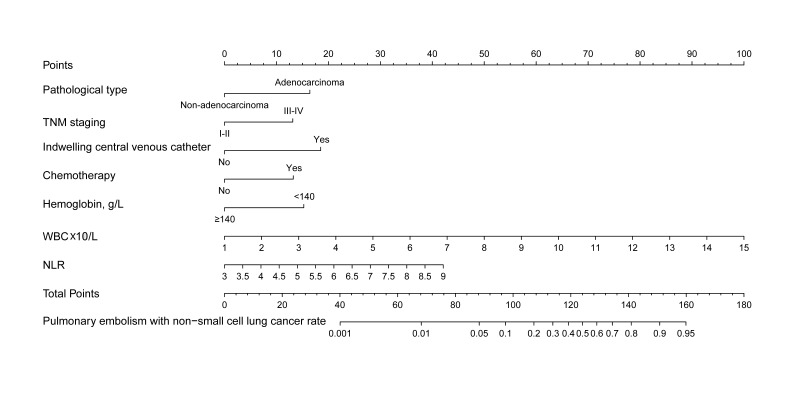
Nomogram to predict the risk of PE in NSCLC.

As the multivariate logistic regression analysis found that seven predictors exhibited statistically significant differences, they were introduced into the final predictive model to develop a quantitative prediction nomogram for discriminating between PE and non-PE in NSCLC patients (Fig.3). The sum of the scores corresponding to each predictive indicator was recorded as the total score. The predicted probability corresponding to the total score represents the risk of PE in patients with NSCLC.

**Fig.4:**
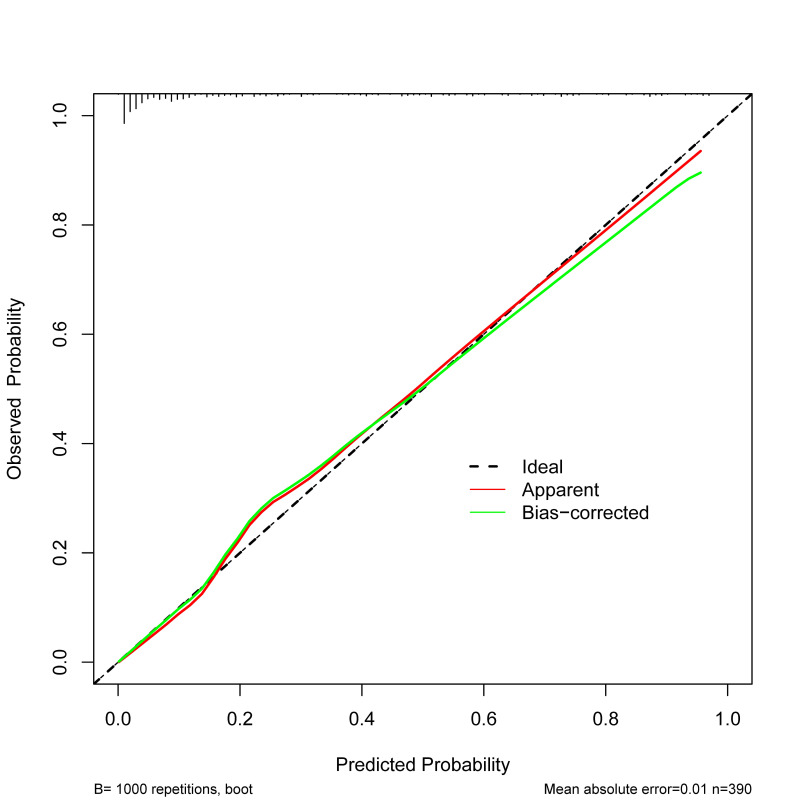
The calibration curve of the nomogram prediction model.

**Fig.5:**
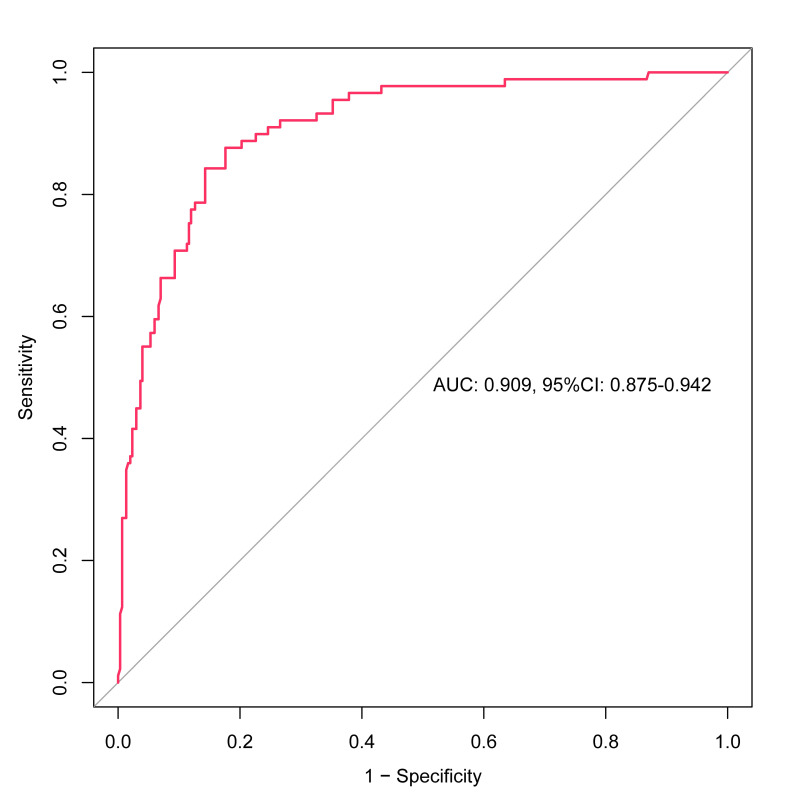
ROC curve and AUC of the predictive model. ROC receiver operating characteristics, AUC area under the curve.

**Fig.6:**
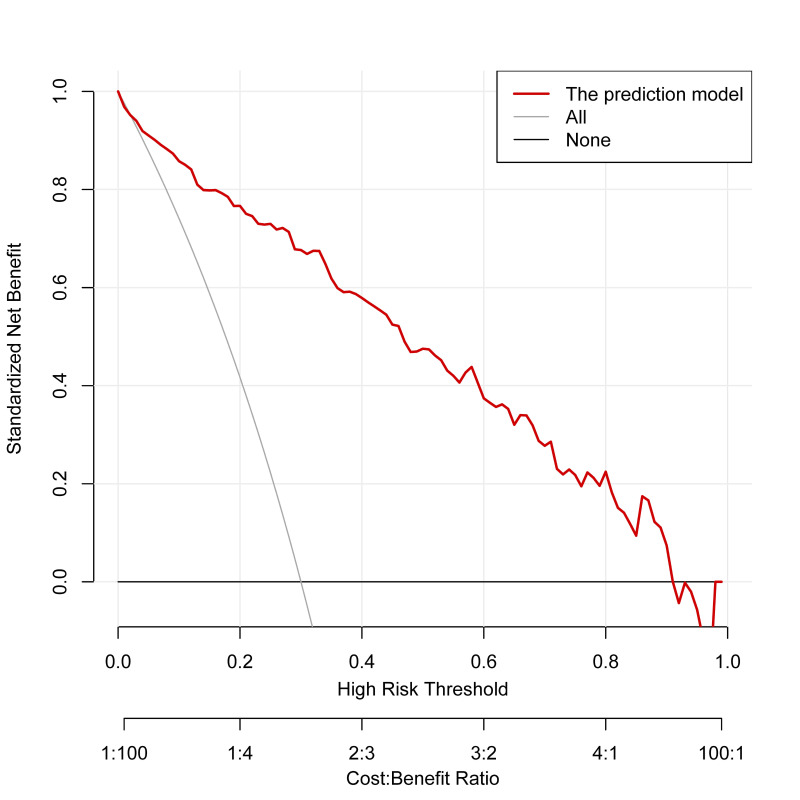
The DCA for the nomogram prediction model. DCA decision curve analysis.

The Hosmer–Lemeshow test showed no statistically significant difference between the predicted values from the nomogram model and the actual observed (χ^2^=3.284, df=8, *P*=0.915). An internal bootstrap validation was conducted using 1000 sampling repetitions, and the C-index was 0.909. Furthermore, the calibration curve derived from the internal bootstrap validation demonstrated a mean absolute error of 0.01 (Fig.4). What’s more, the corrected C-index was 0.900 based on the bootstrapping with optimism-correction. The area under the ROC curve (AUC) was 0.909 (95% CI 0.875–0.942), which indicates that the nomogram model has good discriminative ability (Fig.5). The DCA of the nomogram model was depicted in Fig.6. Within the predicted risk range of 0.01–0.91 for PE in NSCLC patients, it was found that applying preventive measures based on this model resulted in substantially greater net benefits compared to scenarios without any treatment.

## DISCUSSION

We aimed to establish a nomogram for prediction based on NSCLC combined with PE data from the First People’s Hospital of Linping District, Hangzhou in hopes of favoring further clinical practice.

In our study, results showed that seven variables—pathological type, TNM stage, indwelling CVC, chemotherapy, hemoglobin level, WBC count, and NLR—were associated with NSCLC combined with PE. Patients, particularly those with malignant tumors, were prone to abnormalities in coagulation and fibrinolysis within their bodies, which increased their risk of developing thrombosis. Additionally, NSCLC adenocarcinoma cells that could secrete large amounts of mucin which could promote vascular endothelial degeneration and epithelial cell shedding, leading to localized thrombus formation.^11^,^12^ Furthermore, NSCLC cells might release tissue factor and prothrombin activators, which might promote blood coagulation and increase the likelihood of thrombus formation.^13^ In the advanced NSCLC, levels of thrombin and procoagulants were higher, which increased plasminogen activator inhibitor-1, contributing to a greater risk of thrombus formation. Additionally, the advanced NSCLC might compress pulmonary vessels, leading to impaired blood circulation.^14^,^15^ During the insertion of a central venous catheter, there was a risk of vascular wall injury, which could stimulate the vascular endothelial cells and lead to the formation of deep vein thrombosis in the lower limbs, ultimately forming the PE.^16^,^17^ Common chemotherapy drugs could promote the formation of disulfide bonds through the protein disulfide isomerase, thereby increasing the procoagulant activity of cells, leading to vascular wall damage or thrombus formation, thus increasing the risk of PE.^18^,^19^ Hemoglobin was responsible for carrying oxygen to tissues and organs throughout the body. When hemoglobin levels fell below the normal (<140g/L), it indicated insufficient oxygen supply within the body. Moreover, it could lead to increased blood viscosity and reduced blood flow velocity, thereby increasing the risk of thrombus formation.^11^,^20^ The WBC, NLR, PLR, and MLR served as indicators of the systemic inflammatory response and were closely related to the levels of inflammatory cytokines. These markers not only reflected the intensity of inflammation within the body but also impacted on hemostasis. Chronic inflammation could result in coagulopathy, which might predispose individuals to thrombotic events such as PE.^16^,^21^,^22^

The application of predictive models in the medical field has become increasingly widespread, which could help early clinical identification of high-risk populations, early intervention to control adverse outcomes. We synthesized seven independent risk factors and constructed a nomogram model for predicting the quantified individual risk of PE in NSCLC. What’s more, it has been validated that these markers provide good accuracy and discrimination in predicting the risk of PE in NSCLC. Healthcare providers could use the score assigned to each risk factor to individually predict the likelihood of a NSCLC patient developing PE. Clinical practitioners should enhance the screening and management of high-risk factors, minimize the placement of venous catheters, and reduce the use of certain chemotherapeutic agents that might damage vascular endothelium, all in an effort to improve patients’ quality of life. Aggressive treatment of NSCLC through surgery, and radiation therapy could help control disease progression, thereby reducing tumor invasion of pulmonary vessels and lowering the risk of PE. The use of anticoagulant medications could be considered to prevent thrombus formation. Additionally, promoting active lifestyle and exercise can improve circulation, reduce blood stasis, and lower the risk of thrombosis.

Prior research has largely concentrated on the analysis of risk factors associated with PE in NSCLC, yet there has been a notable gap in personalized predictive modeling for these risks. Our study addresses this gap by utilizing a nomogram model to conduct individualized risk assessments, which is significant for the early identification of high-risk populations.

### Limitations:

First, the sample size was small, and this was a single-center retrospective study, which might lead to selection bias. Second, we lacked some crucial risk factors, such as driver mutation gene, and immunotherapy, the inclusion of which could improve the performance of our predictive models. Third, this research performed only internal validation and did not carry out sufficient external validation. Further expansion of sample size, multicenter research, and inclusion of more relevant factors for analysis are necessary to improve the reliability and generalizability of the conclusions.

## CONCLUSION

In our study, we found that NSCLC adenocarcinoma, III–IV TNM staging, indwelling CVC, chemotherapy, low hemoglobin (<140 g/L) and WBC, and NLR increment were the risk factors for NSCLC combined with PE. Moreover, we developed a nomogram prediction model to assess the risk of NSCLC with PE. The nomogram prediction model is expected to identify patients at high risk of NSCLC with PE in clinical practice. However, future research will encompass prospective cohort studies to assess the clinical effectiveness of interventions informed by this model.

### Authors’ contributions:

**XW:** Study design, literature search and manuscript writing.

**XW and ST:** Data collection, data analysis and interpretation. Critical review

**XW:** Manuscript revision and validation and is responsible for the integrity of the study.

All authors have read and approved the final manuscript.
